# The progress made in determining the *Mycobacterium tuberculosis* structural proteome

**DOI:** 10.1002/pmic.201000787

**Published:** 2011-08

**Authors:** Michael Hecker

**Affiliations:** European Molecular Biology Laboratory – Hamburgc/o DESY, Hamburg, Germany

**Keywords:** Microbiology, *Mycobacterium tuberculosis*, Protein structure, Structural proteomics

## Abstract

*Mycobacterium tuberculosis* is a highly infectious pathogen that is still responsible for millions of deaths annually. Effectively treating this disease typically requires a course of antibiotics, most of which were developed decades ago. These drugs are, however, not effective against persistent tubercle bacilli and the emergence of drug-resistant stains threatens to make many of them obsolete. The identification of new drug targets, allowing the development of new potential drugs, is therefore imperative. Both proteomics and structural biology have important roles to play in this process, the former as a means of identifying promising drug targets and the latter allowing understanding of protein function and protein–drug interactions at atomic resolution. The determination of *M. tuberculosis* protein structures has been a goal of the scientific community for the last decade, who have aimed to supply a large amount of structural data that can be used in structure-based approaches for drug discovery and design. Only since the genome sequence of *M. tuberculosis* has been available has the determination of large numbers of tuberculosis protein structures been possible. Currently, the molecular structures of 8.5% of all the pathogen's protein-encoding ORFs have been determined. In this review, we look at the progress made in determining the *M. tuberculosis* structural proteome and the impact this has had on the development of potential new drugs, as well as the discovery of the function of crucial mycobaterial proteins.

## 1 Introduction

Tuberculosis (TB) is an ancient human disease. Evidence of infection with *Mycobacterium tuberculosis*, the causative agent of TB, has been dated to the Neolithic period (7000 BC) and has been observed in Egyptian mummies (2050–500 BC) 1, 2. Reference is made to TB in literature from both ancient China and India dating to 4000–2000 BC as well as in biblical scripture 3. Historically known as consumption in the West, TB was a common disease that led to the death of many historic figures (e.g. John Keats, D. H. Lawrence, George Orwell, Immanuel Kant, Florence Nightingale etc.) and so has particularly influenced European history, and became a theme in art, literature and film. Only in the last century has effective chemotherapeutic treatment been developed against TB. Several antibiotics were discovered in the 1940s and 1950s that led to an initial decline of TB, particularly in developed countries. In the last few decades, however, TB has been resurgent. Driven by poverty, overcrowding and the spread of the human immunodeficiency virus (HIV) in the developing world, TB has been responsible for the death of ∼30 million people worldwide in the last decade 3. A third of the world's population is thought to be infected and 10% of these carry a lifetime risk of developing the disease 3. These worrying facts led the World Health Organization (WHO) to declare TB a global health emergency in 1993 4.

*M. tuberculosis* is highly infectious and is highly adapted to surviving in the host by being able to evade clearance by the immune system and remaining inactive but viable for decades before manifesting as disease. Approximately 9.4 million new cases of TB were recorded in 2009 of which 1.7 million were fatal (World Health Organization, Global tuberculosis control 2010, http://www.who.int/tb/publications/global_report/2010/en/index.html). While prevalence of the disease has declined in the industrialized countries, the disease burden in developing countries remains high. TB is the second major cause of death from an infectious pathogen after HIV 3. In the past century, great strides have been made in treating the disease. There are effective, but complex and often costly, multi-drug treatment regimes that can cure the disease in several months and prevent those who have been in contact with patients from developing the disease. Even in resource-poor settings the treatment strategy DOTS (Directly Observed Therapy, Short Course), which aims to ensure effective therapy, has proven successful in treating TB where properly implemented 3. Where the treatment has been ineffectively implemented, drug-resistant strains of *M. tuberculosis* have arisen that pose a renewed threat to global health. Both multi-drug resistant and extensively drug-resistant *M. tuberculosis* strains have arisen, which are resistant to many of the front-line drugs currently in use 3. There is therefore a clear need for the development of new drugs and the identification of new drug targets. Proteomics and structural biology have a clear role to play in this endeavour. In recent years, several papers have reported the identification of potentially interesting new protein drug targets 5, 6. Understanding the function of these proteins, and indeed also that of existing protein drug targets, often requires detailed knowledge of their structure. In this review, we document the progress that has been made in characterizing the *M. tuberculosis* structural proteome.

## 2 The structural proteome of *M. tuberculosis* in numbers

**Figure 1:**
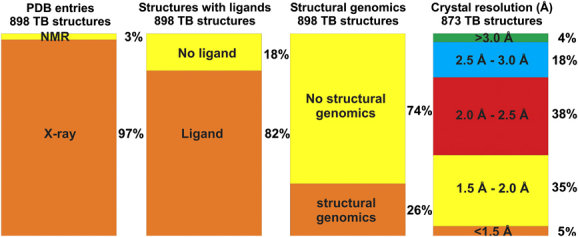
Analysis of *M. tuberculosis* protein structures deposited in the PDB. Shown is the proportion, in percentage of the total number of currently deposited structures (898), that were determined by X-ray crystallography or NMR, the proportion of structures that are ligand-bound, the proportion of structures determined by structural genomics initiatives and the proportion of crystal structures (873) in the different given resolution ranges.

Structural genomics initiatives are responsible for approximately 25% of the determined TB protein structures (Fig. 1). These initiatives, together with the efforts of conventional structural biology groups, have had an enormous impact on the understanding of *M. tuberculosis* biology. The available structural data have already led to the identification of several potential new drug targets (reviewed in [6–8]) and has been helpful in assigning functions to what were previously proteins of unknown function 9, 10.

**Figure 2:**
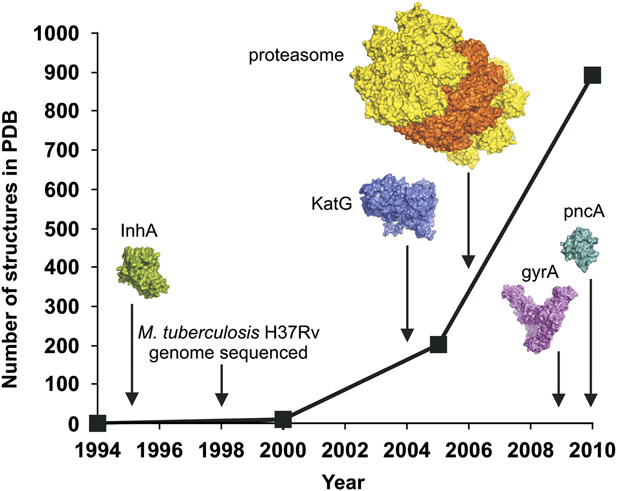
The number of *M. tuberculosis* structures deposited in the PDB to date (December 2010). The first structure was determined in 1994. In 2000 a total of 11 structures had been determined, by 2005 a total of 203 and by 2010 almost 900 structures had been deposited in the PDB. The first publication of the *M. tuberculosis* H37Rv genome sequence is indicated. The structures are those of proteins that are targeted by front-line anti-TB drugs and the proteasome. The year of their deposition in the PDB is indicated with arrows on the time-line. The isoniazide and ethionamide target is inhA (1ENY) 34. Isoniazide is a pro-drug and needs to be activated by KatG (1SJ2) 35. The fluoroquinolones target gyrA (3ILW) and pyrazinamide pncA (3GBC). Rifampin and streptomycin target the ribosome, of which no TB structure has been determined.

One of the biggest breakthroughs in TB research has been the sequencing of the *M. tuberculosis* genome (specifically that of the laboratory strain H37Rv), which was completed in 1998 and re-annotated in 2002 11, 12. The genome sequence offered a great opportunity to understand mycobacterial pathogenesis. Genes important for growth, virulence and persistence are beginning to be identified. Approximately 20% of the structures of proteins involved in virulence and detoxification have been determined and represent the largest proportion of structures solved of any functional class (see below). The sequencing of the genome led to a marked overall increase in the number of structures of *M. tuberculosis* proteins deposited in the Protein Data Bank (PDB) (Fig. 2). The first TB protein structure was determined in 1994 13. In the following 6 years only ten more structures were reported. After the genome sequence of *M. tuberculosis* became publicly available, the number of structures determined after 2000 increased dramatically and at an increasing rate. Initial structural genomics efforts found protein solubility to be a major bottleneck in structure determination 14. Subsequently, methods have been developed that remedied this early problem, such as the development of customized expression strategies for TB proteins in *Escherichia coli* 15 or the use of *Mycobacterium smegmatis* as a heterologous expression strain, as well as the co-expression of proteins using their native-operon structure 16. The increase in determined structures is also no doubt due in part to the development of improved methods for high-throughput crystallography. However, the key event that led to this increase was the availability of genome-wide sequence data as of 1998.

There are currently 69 351 structures in the PDB of which 1.3%, a total of 898, are structures of *M. tuberculosis* proteins. Of these proteins 327 are distinct, which represents 8.5% of the approximately 4000 polypeptide-encoding ORFs in the *M. tuberculosis* genome (Table 1; 12). A complete list of *M. tuberculosis* protein structures deposited in the PDB is given in the Supporting Information Table S1. The predominant method used to determine these structures was X-ray crystallography, which accounts for 873 of the total number of TB structures currently determined (Fig. 1). A further 25 were elucidated by NMR spectroscopy. Most structures are of individual proteins, although many are bound by small molecule ligands such as enzymatic substrates, products, substrate analogues, cofactors and lipids (Fig. 1). Only 11 protein–nucleic acid complexes have been determined and there are very few structures of protein–protein complexes (<10) and none of TB–host protein complexes.

**Table 1:** Functional classification of *M. tuberculosis* proteins and the available structural data for each class

Classa)	Function	ORFsa)	Structures	%Structures/ORFs	Foldsb)	Structures/fold
0	Virulence, detoxification, adaptation	99	20	20.2	15	1.3
1	Lipid metabolism	233	31	13.3	9	3.4
2	Information pathways	229	25	10.9	9	2.8
3	Cell-wall and cell processes	708	31	4.4	11	2.8
6	PE and PPE proteins	170	2	1.2	1	2
7	Intermediate metabolism and respiration	894	143	16.0	35	4.1
8	Proteins of unknown function	272	0	–	–	–
9	Regulatory proteins	189	32	16.9	11	2.9
10	Conserved hypothetical proteins	1051	43	4.1	15	2.9
	Total protein encoding ORFs	3845	327	8.5		

a) Definition of classes and the number of protein-encoding ORFs in the *M. tuberculosis* are as given in Camus et al. 12. Class 4 are RNA-encoding genes and class 5 are insertion sequences and phages and were not included in this analysis.

b) The number of different folds in each functional class are based on the SCOP (http://scop.mrc-lmb.cam.ac.uk/scop/) annotation of the structures given in the Supporting Information Table S1. Only those structures, in total 112, that are annotated in SCOP were considered, which represents 34% of all the distinct protein structures.

*M. tuberculosis* proteins have been broadly classified into ten categories according to their function 12. Table 1 lists these categories and the number of ORFs and structures in each. Classes 4 and 5 are RNA-encoding genes and insertions or phage sequences, respectively. These two classes are not considered in this review. We have restricted ourselves to an analysis of *M. tuberculosis* protein-encoding genes. By far, the largest number of structures to be determined to date is those of enzymes that are responsible for intermediate metabolism and respiration (class 7). This class also contains the largest number of ORFs in the *M. tuberculosis* genome. About 16% of all the ORFs in this category have a representative structure. An equal proportion of structures are available for class 6, the regulatory proteins, and slightly more, approximately 20%, for class 0, the virulence, detoxification and adaptation category. Structures of lipid metabolising proteins, class 1, and those that encode information processing proteins, class 2, are somewhat less well represented in the PDB, but each still cover more then one-tenth of the ORFs in these categories. The categories in which the *M. tuberculosis* structural proteome is under-represented, given the number of ORFs and the progress made in other categories, are the cell wall and cell processes associated proteins (class 3), the proline-glutamate (PE) and proline-proline-glutamate (PPE) proteins (class 6) and the conserved hypothetical proteins (class 10). Only 4.4% of the cell wall and cell processes encoding ORFs have a representative structure, whereas only 4.1% of the conserved hypothetical proteins have a determined structure. The PE and PPE proteins, a family of proteins that are encoded by about 5% of the *M. tuberculosis* genome, have no clearly defined function and only two representative structures have been reported for this class. That represents a mere 1.2% of these genes. It therefore seems, given that structural data has proven useful for the discovery of protein function (see Section 3), that a greater effort should be made to investigate the structures of this poorly understood protein family.

## 3 Structure-based discovery of protein function

Over one-quarter of the *M. tuberculosis* genome encodes conserved hypothetical proteins and 43 of their structures have been determined (Table 1). It is well established that structural motifs and topology are more conserved over evolutionary time than are protein sequences 17. The determination of the structures of these hypothetical proteins may therefore supply some clues as to their biological function. There are several recent examples of the successful use of structural knowledge to predict function.

The function of the enzyme deazaflavin-dependent nitroreductase (Ddn), previously only known as the hypothetical protein Rv3547, was discovered using its homology to Rv1155 and Rv2991 18, 19. The structures of these two proteins were determined by the TB Structural Genomics Consortium and have structural homology to flavin mononucleotide-binding proteins and Nim-proteins that confer resistance to 5-nitroimidazole antibiotics in *Bacteroides* species. Rv3547 is a protein of 151 amino acid residues that had no detectable sequence homology to any other protein of known function 18. Based on its similarity to the two structures, Manjunatha et al. proposed that Rv3547 is a previously uncharacterised class of nitroreductase 18. Subsequently, the enzymatic activity of Rv3547 has been verified 19.

In a similar case, the structure of Rv2175c, originally annotated as a protein of unknown function, revealed that it possesses an original winged helix-turn-helix motif indicative of a DNA-binding activity 20. DNA-binding of Rv2175c was confirmed by fluorescence anisotropy and in electrophoretic mobility shift assays 20. Furthermore, the protein is a substrate of PknL kinase, which negatively regulates Rv2175c's DNA-binding activity by phosphorylating its N-terminus. Its structure, determined by multidimensional NMR, revealed that the N-terminus of the protein is natively unfolded. This N-terminal region seems to be restricted to the species of the *M. tuberculosis* complex, which prompted Cohen-Gonsaud et al. to suggest that this may be a regulatory system unique to these bacteria 20.

*M. tuberculosis* adapts to stress conditions such as a decrease in oxygen by upregulating the dormancy survival regulon, which is associated with the bacterium's ability to enter a nonreplicating persistent state in the host 21. One of the most strongly upregulated ORFs of this regulon is Rv2626c, which encodes a protein called hypoxic response protein 1. Its structure reveals the presence of two disulphide bonds, a structural feature unusual in intracellular proteins, which led to the suggestion that it might be secreted in vivo 22. Tantalizingly, immunogold electron microscopy localization of hypoxic response protein 1 showed it to be extracellular and, most recently, the protein was indeed shown to modulate macrophage effector functions 22, 23. No known signal sequence could be found encoded in Rv2626c. The gene sequence was therefore not predictive of an extracellular function while the structure was. As the above examples and many more reviewed elsewhere 9, 10 demonstrate, structural data can be extremely valuable in assigning function to hypothetical proteins. No doubt the continuing efforts to determine structures of *M. tuberculosis* proteins will lead to greater understanding of this pathogen's biology.

## 4 Structure-based drug discovery

Structural biology can provide molecular details of interactions that are invaluable in understanding the relationships between protein structure and ligand activity, especially when these are drugs or potential drug lead compounds. There are several successful examples of marketable drugs that were developed using protein structure information: the HIV protease inhibitors Viracept 24, Agenerase 25 and Aluviran 26 are such examples. Despite these successes, not many drugs have been developed in this manner. There is, however, growing activity in this area within the TB research community 6. The ongoing efforts to determine large numbers of TB structures are based on the expectation that these are an important source of information for drug discovery programs. Given the dramatically increased wealth of structural data on TB proteins in the last 10 years (Fig. 2), structure-based drug design initiatives have gained momentum, but have as yet to lead to a marketable drug. Most TB protein structures, a total of 688, are of high resolution having been determined with diffraction data better than or equal to 2.5 Å and of these, 350 have resolutions better than 2.0 Å (Fig. 1). Since the ability to determine the structure of a protein–ligand complex at high resolution is an absolute requirement for structure-based drug design, a large number of TB protein structures would therefore be amenable to such methods. Additional information about *M. tuberculosis* structural genomics data from the US is available over the following link: http://www.webtb.org/.

Even though there has been no drug developed against TB as yet using structure-based methods, one recent example of drug-lead development targeting an *E. coli* protein conserved in mycobacteria can serve as an example. A class of amino-oxazoles with antibacterial activity has been identified by researchers of the pharmaceutical company Pfizer as potential drug leads 27, 28. Initially, a class of pyridopyrimidines were identified as selective inhibitors of the *E. coli* biotin carboxylase, the protein that catalyses the first committed step in lipid metabolism 27. These weak-binding small-molecule ligands were then used as building blocks for inhibitors, which through iterative cycles of structure-based drug design that included virtual screening and fragment-based approaches improved the potency of the initial compounds up to 3000-fold 28. Their desirable physicochemical properties were maintained and resulted in competitive inhibitors that target the biotin carboxylase ATP-binding site.

Screening for inhibitors of TB proteins has been performed on several target proteins and in some cases the structure of the proteins are known. This may allow for structure-based drug design of TB specific inhibitors. One set of such potential drug leads has been reported for the carboxyltransferase domain of the *M. tuberculosis* acetyl-CoA carboxylase 29. So far, several computational analyses have led to the prediction of potential drug leads, a process that also relies on the availability of structural data, but this has not yet been followed by actual empirical data. The full potential of structural data to aid the discovery and design of new anti-TB drugs therefore remains to be realized. However, there are some encouraging examples of this. For instance, analysis of the crystal structures of *M. tuberculosis* InhA, the target of the front-line anti-TB drug isoniazide, has been used in structure-based design of potential new drugs 30. Also, structure-based design of DevR inhibitors against nonreplicating *M. tuberculosis* has recently been reported 31.

## 5 Synergy between proteomics and structural genomics

Some recent publications on proteomics and structural biology may represent a new trend in the search for new drug targets 32, 33. Recent proteomics experiments, such as the use of a guinea pig model of aerosol infection to study the *M. tuberculosis* proteome in vivo, have given insight into the expression profile of many of the pathogen's genes 33. Over 500 proteins were identified over the course of infection with several classes such as class 3, cell wall and cell processes, and class 7, intermediate metabolism and respiration, accounting for almost half of these. These indicate new potential targets for structural biology and drug development. More recently, the combination of structural data, modelling and knowledge of drug interactions with proteins was used to determine the drugome of *M. tuberculosis* 32. This study made use of all the structures of TB proteins currently in the PDB and modelled several hundred more to cover approximately 43% of the *M. tuberculosis* proteome. Existing data on protein–drug interactions were used to determine the theoretical proteome-wide drug interaction network, which identified multiple proteins that may already be targeted by existing drugs and that may serve as useful templates for new drugs.

## 6 Concluding remarks

Currently, the molecular structures of 8.5% of the ORF of *M. tuberculosis* have been determined. This has been one of the major achievements within the TB research community during the last decade. The availability of structural data has been invaluable in advancing our understanding of the biology of *M. tuberculosis*. Protein functions could be assigned or re-assigned to many proteins and structural data are being used ever more frequently in the rational design and discovery of new anti-TB drugs. There is continued interest in determining more TB structures and complementation of structural biology with proteomics promises to be a productive new area of research.

## Supporting information

Detailed facts of importance to specialist readers are published as ”Supporting Information”. Such documents are peer-reviewed, but not copy-edited or typeset. They are made available as submitted by the authors.
