# Supplementary material for: The progress made in determining the Mycobacterium tuberculosis structural proteome
Source: Proteomics. 2011 Aug;11(15):3128–33. doi: 10.1002/pmic.201000787 (PMC3345573; doi:10.1002/pmic.201000787)
Supplement: Supplementary file 1 [file pmic0011-3128-SD1.pdf]

PROTEOMICS

Supporting Information for Proteomics

DOI 10.1002/pmic.201000787

Matthias T. Ehebauer and Matthias Wilmanns

The progress made in determining the *Mycobacterium tuberculosis* structural proteome

© 2011 WILEY-VCH Verlag GmbH & Co. KGaA, Weinheim

Table S1. Structures of *M. tuberculosis* proteins deposited in the PDB as of December 2010.

ORF	PDB	Gene names	UniProt	Res.(Å)	Lig.	Sec.[a]	Class[b]	Function annotation	Scop Fold[c]
Rv0005	**3IG0**	gyrB	P0C5C5	2.10			2	DNA gyrase subunit B (EC 5.99.1.3)	
Rv0006	**3ILW**	gyrA	Q07702	1.60	X		2	DNA gyrase subunit A (EC 5.99.1.3)	
Rv0009	**1W74**	ppiA	P65762	2.60			1	Probable peptidyl-prolyl cis-trans isomerase A (EC 5.2.1.8) (Rotamase A)	Cyclophilin-like, 114311
Rv0014c	**3F61**	pknB	P0A5S4	1.80	X		9	Serine/threonine-protein kinase pknB (EC 2.7.11.1)	
Rv0016c	**3LO7**	pbpA	P71586	2.05			3	Penicillin-binding protein A (PBPA)	
Rv0018c	**1TXO**	pstP; mstp	P71588	1.95	X		9	PP2C-family Ser/Thr phosphatase (EC 3.1.3.16)	PP2C-like, 112781
Rv0033	**2CGQ**	acpA; acp-1	P71603	1.83			1	Acyl carrier protein	
Rv0046c	**1GR0**	ino1	P71703	1.95	X		7	Inositol-3-phosphate synthase (IPS) (EC 5.5.1.4)	NAD(P)-binding Rossmann-fold domains, 70379 [14-200], [312-367]; FwdE/GAPDH domain-like 70380 [201-311]
Rv0054	**1UE1**	ssb	P0A610	2.50	X		2	Single-stranded DNA-binding protein (SSB) (Helix-destabilizing protein)	OB-fold, 99243
Rv0058	**2R5U**	dnaB	P71715	1.90	X		2	Replicative DNA helicase (EC 3.6.4.12)	
Rv0062	**1UOZ**	celA1	Q79G13	1.10	X		7	POSSIBLE CELLULASE CELA1 (ENDOGLUCANASE) (EC 3.2.1.4)	7-stranded beta/alpha barrel, 113354
Rv0098	**2PFC**		P64685	2.30	X		10	Conserved hypothetical protein	
Rv0129c	**1DQZ**	fbpC; mpt45	P0A4V4	1.50		X	1	Antigen 85-C (Fibronectin-binding protein C) (EC 2.3.1.-)	alpha/beta-Hydrolases, 34638
Rv0130	**2C2I**		P96807	1.80	X		7	Probable enoyl-CoA hydratase 1 (EC 4.2.1.17)	Thioesterase/thiol ester dehydrase-isomerase, 129670 [2-150]
Rv0137c	**1NWA**	msrA	P0A5L0	1.50			7	Peptide methionine sulfoxide reductase msrA (EC 1.8.4.11)	Ferredoxin-like, 86295
Rv0153c	**1YWF**	ptbB	P96830	1.71	X		9	PHOSPHOTYROSINE PROTEIN PHOSPHATASE PTPB (PTPase) (EC 3.1.3.48)	(Phosphotyrosine protein) phosphatases II, 124144 [4-275]
Rv0216	**2BI0**		P96398	1.90	X		7	Conserved hypothetical protein; double hotdog hydratase	Thioesterase/thiol ester dehydrase-isomerase, 128565 [8-185], 128566 [186-337]
Rv0223c	**3B4W**		P96405	1.80	X		7	Aldehyde dehydrogenase family protein EC 1.2.1.-)	
Rv0233	**3EE4**		P96416	1.90	X		2	R2-like ligand binding oxidase (Ribonucleotide reductase R2 subunit homolog)	
Rv0262c	**1M4I**	aac	P0A5N0	1.50	X		0	Aminoglycoside 2'-N-acetyltransferase (EC 2.3.1.-)	Acyl-CoA N-acyltransferases (Nat), 74454
Rv0287	**2KG7**	esxG	P0A568				3	PE family protein	

Rv0300	**3H87**		O07227	1.49	X		0	Conserved hypothetical protein; Ribbon-helix-helix protein, copG family [PF01402]; PIN domain [PF01850]	
Rv0321	**2QXX**	dcd	O07247	2.00	X		7	Deoxycytidine triphosphate deaminase (EC 3.5.4.13)	
Rv0363c	**3ELF**	fba	P67475	1.31	X		7	Fructose-bisphosphate aldolase (EC 4.1.2.13)	
Rv0371c	**2WEE**		O53706	1.65	X		10	Conserved hypothetical protein	
Rv0390	**2FSX**		P95198	1.80	X		10	Conserved hypothetical protein	
Rv0391	**3NDN**	metZ	P95199	1.85	X		7	O-succinylhomoserine sulfhydrylase (EC 4.2.99.-)	
Rv0407	**3C8N**	fgd1; fgd	P96253	1.90	X		7	F420-dependent glucose-6-phosphate dehydrogenase (EC 1.1.99.34)	
Rv0410c	**2PZI**	pknG	P65728	2.40	X		9	Probable serine/threonine-protein kinase pknG (EC 2.7.11.1)	
Rv0429c	**3E3U**	def	P96275	1.56	X		2	Peptide deformylase (PDF) (EC 3.5.1.88)	
Rv0432	**1PZS**	sodC	P0A608	1.63	X	X	0	Superoxide dismutase [Cu-Zn] (EC 1.15.1.1)	Immunoglobulin-like beta-sandwich, 104397
Rv0440	**1SJP**	groL2; groEL-2	P0A520	3.20			0	60 kDa chaperonin 2	GroEL equatorial domain-like, 112092 [62-134], 112092 [408-514]; GroEL-intermediate domain like, 112094 [135-188], 112094 [373-407]; The "swivelling" beta/beta/alpha domain, 112093 [189-372]
Rv0462	**2A8X**	lpd	P66004	2.40	X		7	Dihydrolipoyl dehydrogenase (EC 1.8.1.4)	
Rv0467	**1F8M**	icl	P0A5H3	1.80	X		7	Isocitrate lyase (EC 4.1.3.1)	TIM beta/alpha-barrel, 29317
Rv0470c	**1L1E**	pcaA;cma3	Q7D9R5	2.00	X		1	Cyclopropane mycolic acid synthase 3 (CMAS) (EC 2.1.1.79)	S-adenosyl-L-methionine-dependent methyltransferases, 73454
Rv0489	**1RII**	gpmA; gpm	P0A5R6	1.70	X		7	2,3-bisphosphoglycerate-dependent phosphoglycerate mutase (EC 5.4.2.1)	Phosphoglycerate mutase-like, 111807
Rv0491	**2OQR**	regX3	Q11156	2.03	X		9	Sensory transduction protein regX3	
Rv0503c	**3HEM**	cmaA2; cma2	P0A5P0	2.39	X		1	Cyclopropane mycolic acid synthase 2 (CMAS) (EC 2.1.1.79)	
Rv0533c	**1U6E**	fabH	P0A574	1.85	X		1	3-oxoacyl-[acyl-carrier-protein] synthase 3 (EC 2.3.1.180)	Thiolase-like, 119568 [-10-174], 119569 [175-317]
Rv0543c	**2KVC**		A5TZS3				10	Conserved hypothetical protein	
Rv0548c	**1Q52**	menB	O06414	1.80			7	Naphthoate synthase (EC 4.1.3.36)	ClpP/crotonase, 95846
Rv0554	**3HSS**	bpoC	O06420	1.90	X		0	Bromoperoxidase, putative (EC 1.11.1.-)	
Rv0603	**2KGY**		O07775				3	POSSIBLE EXPORTED PROTEIN	
Rv0626	**3DBO**		P96917	1.76	X		0	Conserved hypothetical protein; PIN domain [PF01850]; Phd_YefM [PF02604]	
Rv0642c	**2FK8**	mmaA4; hma	Q79FX8	2.00	X		1	Hydroxymycolate synthase MmaA4 (EC 2.1.1.-)	S-adenosyl-L-methionine-dependent methyltransferases, 133648 [22-301]

Rv0644c	**1TPY**	mmaA2; mma2	Q79FX6	2.20	X		1	Cyclopropane mycolic acid synthase MmaA2 (CMAS)	S-adenosyl-L-methionine-dependent methyltransferases, 112608
Rv0674	**3KFW**		O53782	2.50	X		10	Conserved hypothetical protein; PaaX-like protein C-terminal domain [PF08223]	
Rv0733	**2CDN**	adk	P69440	1.90	X		7	Adenylate kinase (AK) (EC 2.7.4.3)	P-loop containing nucleoside triphosphate hydrolases, 130289 [1-181]
Rv0735	**3HUG**	sigL	Q7D9D3	2.35	X		2	RNA polymerase sigma factor	
Rv0757	**2PMU**		Q7D9B8	1.7	X		9	DNA-binding response regulator	
Rv0760c	**2A15**		P71817	1.68	X		10	Conserved hypothetical protein	Cystatin-like, 125973 [5-136]
Rv0764c	**2CIB**	cyp51	P0A512	1.50	X		7	Lanosterol 14-alpha demethylase (EC 1.14.13.70)	Cytochrome P450, 146398 [5-449]
Rv0793	**1Y0H**		O86332	1.60	X		10	Conserved hypothetical protein	Ferredoxin-like, 116303
Rv0802c	**2VZY**		O06632	2.00	X		10	Conserved hypothetical protein	
Rv0805	**3IB7**	icc	O06629	1.60	X		10	Conserved hypothetical protein; Calcineurin-like phosphoesterase [PF00149]	
Rv0813c	**2FWV**		O53827	1.70	X		10	Conserved hypothetical protein	
Rv0819	**1P0H**	mshD	O53831	1.60	X		7	Mycothiol acetyltransferase (MSH acetyltransferase) (EC 2.3.1.189)	Acyl-CoA N-acyltransferases (Nat), 93866
Rv0844c	**3EUL**	narL	O53856	1.90	X		9	Probable transcriptional regulatory protein NarL	
Rv0855	**2G04**	far	O53867	2.70			1	Fatty acid-CoA racemase (EC 5.1.-.-)	
Rv0858c	**2O0R**		O53870	2.00	X		7	Aminotransferase, class I (EC 2.6.1.-)	
Rv0865	**2G4R**	mog	O53877	1.92	X		7	Molybdopterin biosynthesis Mog protein	
Rv0884c	**2FYF**	serC	P63514	1.50	X		7	Putative phosphoserine aminotransferase (EC 2.6.1.52)	
Rv0899	**2KGS**	ompA	P65593				3	Outer membrane protein A; porin-like	
Rv0902c	**1YSR**	prrB	P0A5Z8	1.78	X	X	9	Sensor-type histidine kinase prrB (EC 2.7.13.3)	ATPase domain of HSP90 chaperone/DNA topoisomerase II/histidine kinase, 123981 [299-446]
Rv0903c	**1YS7**	prrA	P0A5Z6	1.58	X		9	Transcriptional regulatory protein prrA	Flavodoxin-like, 123962 [7-127]; DNA/RNA-binding 3-helical bundle, 123961 [128-233]
Rv0905	**3HE2**	echA6	P64014	2.30	X		1	Probable enoyl-CoA hydratase echA6 (EC 4.2.1.17)	
Rv0931c	**1RWI**	pknD	O05871	1.80	X		9	Serine/threonine-protein kinase pknD (EC 2.7.11.1)	6-bladed beta-propeller, 97987
Rv0934	**1PC3**	pstS1; phoS1	P15712	2.16	X	X	3	Phosphate-binding protein; Bacterial extracellular solute-binding protein [PF01547]	Periplasmic binding protein-like II, 104102
Rv0938	**2IRU**		P71571	1.65			7	Putative DNA ligase-like protein	
Rv0946c	**2WU8**	pgi	P64192	2.25	X		7	Glucose-6-phosphate isomerase (GPI) (EC 5.3.1.9)	
Rv0948c	**2VKL**		P64767	1.65	X		10	Conserved hypothetical protein	
Rv0956	**3DA8**	purN	P71554	1.30	X		7	Phosphoribosylglycinamide formyltransferase (EC 2.1.2.2)	

Rv0967	**2HH7**	csoR	P71543	2.55	X		10	Conserved hypothetical protein; Copper-sensing transcriptional repressor CsoR	
Rv0983	**1Y8T**	pepD	O53896	2.00	X		7	SERINE PROTEASE PEPD (EC 3.4.21.-)	Trypsin-like serine proteases, 122764 [6-226]; PDZ domain-like, 122763 [227-314]
Rv0985c	**2OAR**	mscL	A5U127	3.50	X		3	Large-conductance mechanosensitive channel	
Rv1009	**3EO5**	rpfB	O05594	1.83		X	3	Probable resuscitation-promoting factor rpfB	
Rv1014c	**2Z2I**	pth	P65865	1.98			7	Peptidyl-tRNA hydrolase (PTH) (EC 3.1.1.29)	
Rv1018c	**3DK5**	glmU	A5U161	2.23	X		3	Bifunctional protein [Includes: UDP-N-acetylglucosamine pyrophosphorylase (EC 2.7.7.23); Glucosamine-1-phosphate N-acetyltransferase (EC 2.3.1.157)]	
Rv1070c	**3H81**	echA8	P64016	1.80	X		1	Probable enoyl-CoA hydratase echA8 (EC 4.2.1.17)	
Rv1086	**2VG1**		O53434	1.70	X		3	Short-chain Z-isoprenyl diphosphate synthase (EC 2.5.1.68)	
Rv1092c	**3AEZ**	coaA	P63810	2.20	X		7	Pantothenate kinase (EC 2.7.1.33)	
Rv1093	**3H7F**	glyA1; glyA	O53441	1.50	X		7	Serine hydroxymethyltransferase 1 (EC 2.1.2.1)	
Rv1094	**1ZA0**	desA2	O53442	2.00	X		1	Putative acyl-[acyl-carrier-protein] desaturase desA2 (DES) (EC 1.14.19.-)	Ferritin-like, 124774 [8-274]
Rv1098c	**3NO9**	fumC; fum	O53446	2.48			7	Fumarate hydratase class II (EC 4.2.1.2)	
Rv1131	**3HWK**	gltA1; gltA-3	O08395	2.30	X		7	Citrate synthase (EC 2.3.3.1)	
Rv1143	**2GCI**	mcr	O06543	1.60	X		1	2-methylacyl-CoA racemase (EC 5.1.99.4)	CoA-transferase family III (CaiB/BaiF), 134983 [2-360]
Rv1155	**2AQ6**		O06553	1.70	X		10	Putative pyridoxine/pyridoxamine 5'-phosphate oxidase (EC 1.4.3.5)	Split barrel-like, 127156 [5-147]
Rv1170	**1Q74**	mshB	O50426	1.70	X		7	1D-myo-inositol 2-acetamido-2-deoxy-alpha-D-glucopyranoside deacetylase (EC 3.5.1.103)	LmbE-like,96019
Rv1201c	**3FSY**		O05302	1.97	X		7	Tetrahydrodipicolinate N-succinyltransferase (putative) (EC 2.3.1.117)	
Rv1207	**2VP8**	folP2	P64139	2.64	X		7	Inactive dihydropteroate synthase 2 (DHPS 2)	
Rv1208	**3E26**		O05309	2.50			10	Putative uncharacterized protein	
Rv1256c	**2UUQ**	cyp130	Q11062	1.46	X		7	Putative cytochrome P450 130 (EC 1.14.-.-)	
Rv1264	**2EV1**		Q11055	1.60	X		7	pH-sensitive adenylate cyclase Rv1264 (EC 4.6.1.1)	
Rv1267c	**2FF4**	embR	P14737	1.90	X		9	Probable regulatory protein embR	DNA/RNA-binding 3-helical bundle, 133367 [10-104]; alpha-alpha superhelix, 133368 [105-283]; SMAD/FHA domain, 133369 [284-382]
Rv1284	**1YLK**		P64797	2.00	X		10	Putative carbonate dehydratase-like protein (EC 4.2.1.-)	
Rv1293	**2O0T**	lysA	P0A5M4	2.33	X		7	Diaminopimelate decarboxylase (EC 4.1.1.20)	

Rv1295	**2D1F**	thrC	P66902	2.50	X		7	Threonine synthase (EC 4.2.3.1)	
Rv1314c	**2G2D**		P64803	2.00			7	Cob(I)yrinic acid a,c-diamide adenosyltransferase (EC 2.5.1.17)	
Rv1326c	**3K1D**	glgB	Q10625	2.33			7	1,4-alpha-glucan-branching enzyme (EC 2.4.1.18)	
Rv1335	**3DWG**	cysO; cfp10A	P0A646	1.53	X		10	Conserved hypothetical protein; Sulfur carrier protein CysO (9.5 kDa culture filtrate antigen cfp10A)	
Rv1336	**3FGP**	cysM	P63873	2.05	X		7	O-phosphoserine sulfhydrylase (EC 2.5.1.65)	
Rv1340	**3B4T**	rph; rphA	Q10628	2.10	X		2	Ribonuclease PH (EC 2.7.7.56)	
Rv1347c	**1YK3**	mbtK	P64819	2.20	X		10	Conserved hypothetical protein; Lysine N-acyltransferase (EC 2.3.1.-)	Acyl-CoA N-acyltransferases (Nat), 123493 [10-207]
Rv1364c	**3K3C**		Q11034	1.62	X		10	Conserved hypothetical protein	
Rv1372	**1TED**		Q7D8I1	2.25	X		10	Conserved hypothetical protein	Thiolase-like, 106807
Rv1379	**1W30**	pyrR	P65941	1.90			9	Bifunctional protein pyrR [Includes: Pyrimidine operon regulatory protein; Uracil phosphoribosyltransferase (UPRTase) (EC 2.4.2.9)]	PRTase-like, 114118
Rv1389	**1ZNW**	gmk	P0A5I4	2.10			7	Guanylate kinase (EC 2.7.4.8)	P-loop containing nucleoside triphosphate hydrolases, 125412 [20-201]
Rv1404	**2NYX**		P71672	2.30	X		9	Probable transcriptional regulatory protein	
Rv1411c	**3MH9**	lprG; lpp-27	P0A5I8	1.79			3	Lipoprotein lprG (Antigen P27)	
Rv1416	**2C92**	ribH	P66034	1.60	X		7	6,7-dimethyl-8-ribityllumazine synthase (Lumazine synthase) (EC 2.5.1.9)	
Rv1438	**3GVG**	tpiA; tpi	P66940	1.55	X		7	Triosephosphate isomerase (EC 5.3.1.1)	
Rv1445c	**3ICO**	pgl; devB	P63338	2.15	X		7	6-phosphogluconolactonase (EC 3.1.1.31)	
Rv1477	**3NE0**	RipA	O53168	1			0	Hypothetical invastion protein	
Rv1483	**1UZM**	fabG; fabG1	P0A5Y4	1.49	X		1	3-oxoacyl-[acyl-carrier-protein] reductase (EC 1.1.1.100)	NAD(P)-binding Rossmann-fold domains, 119811 [9-245]
Rv1484	**2H7I**	inhA	P0A5Y6	1.62	X		1	Enoyl-[acyl-carrier-protein] reductase [NADH] (EC 1.3.1.9)	NAD(P)-binding Rossmann-fold domains, 136215 [2-269]
Rv1496	**3P32**		P63577	1.9	X		3	Probable GTPase Rv1496/MT1543 (EC 3.6.-.-)	
Rv1542c	**2GKM**	glbN	P0A592	1.73	X		7	Group 1 truncated hemoglobin glbN	
Rv1568	**3LV2**	bioA	P0A4X6	2.18	X		7	Adenosylmethionine-8-amino-7-oxononanoate aminotransferase (EC 2.6.1.62)	
Rv1570	**3FGN**	bioD	O06620	1.85			7	Dethiobiotin synthetase (EC 6.3.3.3)	
Rv1596	**1QPR**	nadC	O06594	2.45	X		7	Nicotinate-nucleotide pyrophosphorylase [carboxylating] (EC 2.4.2.19)	alpha/beta-Hammerhead, 38609 [2-116]; TIM beta/alpha-barrel, 29573 [117-285]
Rv1612	**2O2E**	trpB	P66984	2.20			7	Tryptophan synthase beta chain (EC 4.2.1.20)	
Rv1625c	**1YK9**	cya	P0A4Y0	2.70			7	Adenylate cyclase (EC 4.6.1.1)	

Rv1626	**1S8N**	pdtaR	O06143	1.48	X		9	Probable transcriptional regulatory protein pdtaR	Flavodoxin-like, 105374
Rv1636	**1TQ8**		O06153	2.40	X		10	Conserved hypothetical protein	Adenine nucleotide alpha hydrolase-like, 107204
Rv1652	**2NQT**	argC	P63562	1.58	X		7	N-acetyl-gamma-glutamyl-phosphate reductase (AGPR) (EC 1.2.1.38)	
Rv1653	**3IT4**	argJ	P63571	1.70	X		7	Arginine biosynthesis bifunctional protein ArgJ: Glutamate N-acetyltransferase (EC 2.3.1.35) Ornithine acetyltransferase (EC 2.3.1.1)]	
Rv1654	**2AP9**	argB	P0A4Y6	2.80	X		7	Acetylglutamate kinase (EC 2.7.2.8)	
Rv1656	**2P2G**	argF	P0A5M8	2.70	X		7	Ornithine carbamoyltransferase (EC 2.1.3.3)	
Rv1657	**2ZFZ**	argR; ahrC	P0A4Y8	1.85	X		9	Arginine repressor	
Rv1677	**1ZZO**	dsbF	O53924	1.60		X	3	PROBABLE LIPOPROTEIN; Redoxin [PF08534]	Thioredoxin fold, 125913 [45-178]
Rv1689	**2JAN**	tyrS	P67611	2.90			2	Tyrosyl-tRNA synthetase (EC 6.1.1.1)	
Rv1695	**1U0T**	ppnK	P0A5S6	2.30			7	Inorganic polyphosphate/ATP-NAD kinase (EC 2.7.1.23)	NAD kinase/diacylglycerol kinase-like, 107567
Rv1700	**1MK1**		O33199	2.00	X		2	MutT/nudix family protein	Nudix, 91306
Rv1710	**2Z99**	scpB	O33209	2.30			10	Conserved hypothetical protein	
Rv1743	**2H34**	pknE	P72001	2.80	X		9	Serine/threonine-protein kinase (EC 2.7.11.1)	
Rv1761c	**2K3M**		O06796		X		3	Hypothetical exported protein	
Rv1825	**3GMG**		P64895	1.50			10	Conserved hypothetical protein	
Rv1826	**3HGB**	gcvH	Q50607	1.75			7	Glycine cleavage system H protein	
Rv1827	**2KFU**		P64897		X		10	Conserved hypothetical protein	
Rv1837c	**1N8I**	glcB	P0A5J4	2.10	X		7	Malate synthase G (EC 2.3.3.9)	TIM beta/alpha-barrel, 80297
Rv1846c	**2G9W**	blaI	P95163	1.80	X		9	Transcriptional regulator BlaI	DNA/RNA-binding 3-helical bundle, 147097 [3-124]
Rv1848	**2FVH**	ureA	P0A676	1.80			7	Urease subunit gamma (EC 3.5.1.5)	
Rv1873	**2JEK**		O07756	1.38	X		10	Conserved hypothetical protein	Rv1873-like, 138286 [6-145]
Rv1876	**2WTL**	bfr; bfrA	P63697	2.59	X		7	Bacterioferritin	
Rv1885c	**2FP1**		O07746	1.55	X	X	10	Conserved hypothetical protein; Chorismate mutase-related protein [PF01817]	Chorismate mutase II, 133891 [35-199]
Rv1886c	**1F0N**	fbpB	P0C5B9	1.80	X	X	1	Antigen 85-B (Antigen 85 complex B) (EC 2.3.1.-)	alpha/beta-Hydrolases, 34636
Rv1900c	**1YBT**	lipJ	O07732	2.31	X		7	PROBABLE LIGNIN PEROXIDASE	
Rv1908c	**2CCA**	katG	Q08129	2.00	X		0	Catalase-peroxidase (CP) (EC 1.11.1.6) (EC 1.11.1.7) (Peroxidase/catalase)	Heme-dependent peroxidases, 130222 [26-435], 130223 [436-720]
Rv1926c	**1LMI**	mpt63	P0A5Q2	1.50		X	3	Immunogenic protein MPT63 (16 kDa immunoprotective extracellular protein)	Immunoglobulin-like beta-sandwich, 78098
Rv1932	**1XVQ**	tpx	P66952	1.75	X		0	Probable thiol peroxidase (EC 1.11.1.-)	Thioredoxin fold, 116094

Rv1938	**2ZJF**	ephB	P95276	2.40	X		0	Epoxide hydrolase (EC 3.3.2.3)	
Rv1941	**3GVC**		P95273	2.45			7	Oxidoreductase (EC 1.-.-.-); short chain dehydrogenase [PF00106]	
Rv1980c	**2HHI**	mpt64	P0A5Q4				3	Immunogenic protein MPT64	
Rv1985c	**3ISP**		P67665	2.70			9	Uncharacterized HTH-type transcriptional regulator	
Rv1994c	**2JSC**	cmtR	P67731		X		9	HTH-type transcriptional regulator CmtR	
Rv2002	**1NFF**	fabG3	P69167	1.80	X	X	1	3-alpha-(or 20-beta)-hydroxysteroid dehydrogenase (EC 1.1.1.53)	NAD(P)-binding Rossmann-fold domains, 80457
Rv2027c	**2VZW**	dosT	O53473	2.30	X		9	Hypoxia sensor histidine kinase response regulator dosT	
Rv2043c	**3GBC**	pncA	Q50575	2.20	X		7	PYRAZINAMIDASE/NICOTINAMIDAS(EC 3.5.1.-)	
Rv2068c	**3M6B**	blaA; blaC	P0C5C1	1.30	X	X	7	Beta-lactamase (EC 3.5.2.6)	
Rv2069	**2O7G**	sigC	P66809	2.70	X		2	Probable RNA polymerase sigma-C factor	
Rv2074	**2ASF**		Q10682	1.60	X		10	Conserved hypothetical protein	Split barrel-like, 127253 [11-135]
Rv2109c	**2FHH**	prcA	O33244	2.99	X		7	Proteasome subunit alpha	
Rv2110c	**2JAY**	prcB	O33245	1.99			7	Proteasome subunit beta (EC 3.4.25.1)	
Rv2111c	**3M91**	PUP	O33246	1.80			7	Proteasome-associated ATPase; Pup$_{21–64}$–Mpa$_{46–96}$ complex	
Rv2115c	**3FP9**	mpa	P63345	2.00			3	Proteasome-associated ATPase (AAA ATPase forming ring-shaped complexes)	
Rv2118c	**1I9G**		O33253	1.98	X		7	POSSIBLE RNA METHYLTRANSFERASE (EC 2.1.1.-)	S-adenosyl-L-methionine-dependent methyltransferases, 62090
Rv2121c	**1NH8**	hisG	P60759	1.80	X		7	ATP phosphoribosyltransferase (EC 2.4.2.17)	Periplasmic binding protein-like II, 80507 [1-210]; Ferredoxin-like, 80508 [211-284]
Rv2122c	**1Y6X**	hisE	P0A5B1	1.25	X		7	Phosphoribosyl-ATP pyrophosphatase (EC 3.6.1.31)	all-alpha NTP pyrophosphatases, 122676 [7-93]
Rv2150c	**1RQ2**	ftsZ	P64170	1.86	X		3	Cell division protein	Tubulin nucleotide-binding domain-like, 105048 [8-205]; Bacillus chorismate mutase-like, 105049 [206-312]
Rv2158c	**2WTZ**	murE	P65477	3.00	X		3	UDP-N-acetylmuramoyl-L-alanyl-D-glutamate-2,6-diaminopimelate ligase (EC 6.3.2.13)	
Rv2175c	**2KFS**		O53509				9	Conserved hypothetical regulatory protein	
Rv2178c	**3KGF**	aroG	O53512	2.00	X		7	3-deoxy-D-arabino-heptulosonate 7-phosphate synthase AroG (EC 2.5.1.54)	
Rv2192c	**2BPQ**	trpD	P66992	1.90	X		7	Anthranilate phosphoribosyltransferase (EC 2.4.2.18)	
Rv2202c	**2PKF**	adoK; cbhK	P83734	1.50			7	Adenosine kinase (EC 2.7.1.20)	
Rv2210c	**3HT5**	ilvE	Q10399	1.90	X		7	Branched-chain-amino-acid aminotransferase (EC	

								2.6.1.42)	
Rv2217	**1W66**	lipB	Q10404	1.08	X		7	Octanoyltransferase (EC 2.3.1.181)	Class II aaRS and biotin synthetases, 120652 [1-216]
Rv2220	**2BVC**	glnA1; glnA	P0A590	2.10	X		7	Glutamine synthetase 1 (EC 6.3.1.2)	beta-Grasp (ubiquitin-like), 129243 [5-104]; Glutamine synthetase/guanido kinase, 129244 [105-478]
Rv2225	**1OY0**	panB	P0A5Q8	2.80	X		7	3-methyl-2-oxobutanoate hydroxymethyltransferase (EC 2.1.2.11)	TIM beta/alpha-barrel, 87543
Rv2228c	**3HST**		P64955	2.25	X		10	Conserved hypothetical protein	
Rv2234	**1U2P**	ptpA	P65716	1.90	X		9	low molecular weight protein-tyrosine-phosphatase (PTPase) (EC 3.1.3.48)	
Rv2238c	**1XVW**		P65688	1.90	X		0	Thioredoxin reductase (EC 1.11.1.15)	Thioredoxin fold, 122387 [1-153]
Rv2243	**2QC3**	fabD	P63458	2.30	X		1	Malonyl CoA-acyl carrier protein transacylase (EC 2.3.1.39)	
Rv2244	**1KLP**	acpM	P0A4W6				1	Meromycolate extension acyl carrier protein	Acyl carrier protein-like, 72721
Rv2245	**2WGE**	kasA	P63454	1.80	X		1	3-oxoacyl-[acyl-carrier-protein] synthase 1 (EC 2.3.1.41)	
Rv2246	**2GP6**	kasB	P63456	2.40			1	3-oxoacyl-[acyl-carrier-protein] synthase 2 (EC 2.3.1.41)	
Rv2266	**2WM5**	cyp124	P0A516	1.50	X		7	Putative cytochrome P450 124 (EC 1.14.-.-)	
RV2275	**2X9Q**		Q50688	2.02	X		10	Conserved hypothetical protein	
Rv2276	**1N40**	cyp121	P0A514	1.06	X		7	Cytochrome P450 121 (EC 1.14.-.-)	Cytochrome P450, 79977
Rv2302	**2A7Y**		P64983				10	Conserved hypothetical protein	SH3-like barrel, 126382 [1-80]
Rv2334	**2Q3B**	cysK1; cysK	P0A534	1.80	X		7	O-acetylserine sulfhydrylase (EC 2.5.1.47)	
Rv2346c	**3OGI**		P95243	2.55	X		10	Putative ESAT-6-like protein 6	
Rv2359	**2O03**	furB; fur-2	O05839	2.70	X		9	Ferric uptake regulation protein	
Rv2361c	**2VG3**	uppS	P60479	1.80	X		3	Undecaprenyl pyrophosphate synthase (EC 2.5.1.31)	
Rv2377c	**2KHR**	mbtH	O05821				1	Protein mbtH	
Rv2386c	**2G5F**	mbtI; trpE2	Q7D785	1.80	X	X	1	Isochorismate synthase/isochorismate-pyruvate lyase (EC 4.1.3.-) (EC 5.4.4.2)	ADC synthase, 134654 [15-449]
Rv2391	**1ZJ8**	sir; nirA	P71753	2.80	X		7	Sulfite reductase [ferredoxin] (EC 1.8.7.1)	Ferredoxin-like, 146004 [10-161], 146003 [327-406]; Nitrite and sulphite reductase 4Fe-4S domain-like, 146006 [162-326], 146005 [407-555]
Rv2428	**2BMX**	ahpC	Q7BHK8	2.40	X		0	Alkyl hydroperoxide reductase subunit C (EC 1.11.1.15)	Thioredoxin fold, 128817 [2-170]
Rv2429	**1GU9**	ahpD	P0A5N4	1.90	X		0	Alkyl hydroperoxide reductase (EC 1.11.1.15)	AhpD-like, 65540

Rv2430c	**2G38**	PPE41	Q79FE1	2.20	X		6	PPE FAMILY PROTEIN	Ferritin-like, 134550 [2-174]
Rv2431c	**2G38**	PE25	Q7D756	2.20	X		6	PE family protein	Ferritin-like, 134549 [8-84]
Rv2436	**3GO6**	rbsK	P71913	1.98	X		7	RIBOKINASE (EC 2.7.1.15)	
Rv2438c	**3DLA**	nadE	P0A5L6	2.35	X		7	Glutamine-dependent NAD(+) synthetase (EC 6.3.5.1)	
Rv2445c	**1K44**	ndkA; ndk	P84284	2.60			7	Nucleoside diphosphate kinase (EC 2.7.4.6)	Ferredoxin-like, 72033
Rv2447c	**2VOS**	folC	O53174	2.00	X		7	Folylpolyglutamate synthase (EC 6.3.2.17)	
Rv2461c	**2CBY**	clpP1; clpP	P0A526	2.60			7	ATP-dependent Clp protease proteolytic subunit 1 (EC 3.4.21.92)	ClpP/crotonase' 130202 [15-193]
Rv2465c	**2VVP**	rpiB	Q79FD7	1.65	X		7	Ribose-5-phosphate isomerase B (EC 5.3.1.6)	Ribose/Galactose isomerase RpiB/AlsB, 153645 [3-158]
Rv2470	**2QRW**	glbO	P0A595	1.93	X		7	Group 2 truncated hemoglobin glbO	
Rv2495c	**3L60**	pdhC	O06159	2.00	X		7	probable dihdrolipoamide S-acetyltransferase E2 component (EC 2.3.1.12)	
Rv2498c	**1U5H**	citE	O06162	1.65	X		7	Citrate lyase subunit beta-like protein (EC 4.1.-.-)	TIM beta/alpha-barrel, 113037
Rv2523c	**3NE3**	acpS	P0A4W8	1.90			1	Holo-[acyl-carrier-protein] synthase (EC 2.7.8.7)	
Rv2533c	**1EYV**	nusB	P95020	1.60	X	X	2	N utilization substance protein B homolog	NusB-like, 18446
Rv2537c	**1H05**	aroQ; aroD	P0A4Z6	1.50	X		7	3-dehydroquinate dehydratase (EC 4.2.1.10)	Flavodoxin-like, 76433
Rv2539c	**2IYV**	aroK	P0A4Z2	1.35	X		7	Shikimate kinase (EC 2.7.1.71)	P-loop containing nucleoside triphosphate hydrolases, 137812 [2-166]
Rv2540c	**2QHF**	aroC; aroF	P63611	1.65	X		7	Chorismate synthase (EC 4.2.3.5)	
Rv2543	**2V7S**	lppA	P95010	1.96	X		3	Putative lipoprotein lppA	
Rv2579	**2QVB**	dhaA	Q50642	1.19	X		7	Haloalkane dehalogenase 3 (EC 3.8.1.5)	
Rv2593c	**2ZTD**	ruvA	P66744	2.40	X		2	Holliday junction ATP-dependent DNA helicase (EC 3.6.4.12)	
Rv2607	**2A2J**	pdxH	P65682	2.50	X		7	Pyridoxine/pyridoxamine 5'-phosphate oxidase (EC 1.4.3.5)	Split barrel-like, 126033 [24-224]
Rv2623	**3CIS**		O06189	2.90	X		10	Conserved hypothetical protein; Universal stress protein	
Rv2626c	**1Y5H**	HRP1	O06186	1.50	X	X	0	Hypoxic response protein 1	CBS-domain pair, 144595 [2-124]
Rv2632c	**2FGG**		P65033	2.30	X		10	Conserved hypothetical protein	dsRBD-like, 133438 [4-87]
Rv2697c	**3HZA**	dut	P0A552	1.20	X		7	Deoxyuridine 5'-triphosphate nucleotidohydrolase (EC 3.6.1.23)	
Rv2701c	**2Q74**	suhB	P65165	2.60			0	Inositol-1-monophosphatase (EC 3.1.3.25)	
Rv2704	**3I7T**		O07205	1.93	X		10	Conserved hypothetical protein	
Rv2711	**2ISY**	ideR; dtxR	P0A672	1.96	X		9	Iron-dependent repressor	DNA/RNA-binding 3-helical bundle, 137612 [2-64]; Iron-dependent repressor protein, dimerization domain, 137613 [65-140]

Rv2714	**2WAM**		O07213	2.60	X		10	Conserved hypothetical protein	
Rv2717c	**2FR2**		O07216	1.50			10	Conserved hypothetical protein	Lipocalins, 133963 [4-164]
Rv2726c	**3FVE**	dapF	P63897	2.60	X		7	Diaminopimelate epimerase (EC 5.1.1.7)	
Rv2737c	**2IN0**	recA	P0A5U4	1.60			2	Recombinase A	
Rv2740	**2BNG**		Q7TY00	2.50	X		0	Limonene-1,2-epoxide hydrolase catalytic domain [PF07858]	Cystatin-like, 146156 [13-144]
Rv2753c	**1XXX**	dapA	P63945	2.28	X		7	Dihydrodipicolinate synthase (EC 4.2.1.52)	TIM beta/alpha-barrel, 122433 [5-300]
Rv2754c	**3GWC**	thyX	P66930	1.90	X		7	Thymidylate synthase (EC 2.1.1.148)	
Rv2763c	**1DF7**	folA; dfrA	P0A546	1.70	X		7	Dihydrofolate reductase (EC 1.5.1.3)	Dihydrofolate reductase-like, 34885
Rv2773c	**1P9L**	dapB	P72024	2.30	X		7	Dihydrodipicolinate reductase (EC 1.3.1.26)	NAD(P)-binding Rossmann-fold domains, 94393 [1-105], 94393 [215-245]; FwdE/GAPDH domain-like, 94394 [106-214]
Rv2780	**2VHW**	ald	P30234	2.00	X		7	Alanine dehydrogenase (EC 1.4.1.1)	
Rv2793c	**1SGV**	truB	P62190	1.90			2	tRNA pseudouridine synthase B (EC 5.4.99.-)	Pseudouridine synthase, 98859 [3-235]; PUA domain-like, 98858 [236-292]
Rv2827c	**1ZEL**		Q7D6H9	1.93	X		10	Conserved hypothetical protein	DNA/RNA-binding 3-helical bundle, 145994 [1-82]; Rv2827c C-terminal domain-like, 145995 [83-294]
Rv2841c	**2ASB**	nusA	2ASB	1.50	X		2	Transcription elongation protein nusA	OB-fold, 127244 [108-183]; Alpha-lytic protease prodomain-like, 127245 [184-262], 127246 [263-329]
Rv2844	**2IB0**		O05815	2.00			10	Conserved hypothetical protein	Ferritin-like, 147597 [17-158]
Rv2861c	**3IU7**	map; mapB	P0A5J2	1.40	X		7	Methionine aminopeptidase (EC 3.4.11.18)	
Rv2865	**3G5O**		O33347	2.00	X		0	Conserved hypothetical protein; possible antitoxin	
Rv2870c	**2C82**	dxr	P64012	1.90	X		7	1-deoxy-D-xylulose 5-phosphate reductoisomerase (EC 1.1.1.267)	
Rv2874	**2HYX**	dipZ	Q10801	1.90			7	Protein dipZ	
Rv2875	**1NYO**	mpt70	P0A668			X	3	Immunogenic protein MPT70	FAS1 domain, 92345
Rv2878c	**1LU4**	mpt53; mpb53	P0A618	1.12		X	3	Soluble secreted antigen MPT53	Thioredoxin fold, 91124
Rv2882c	**1WQG**	frr	P66734	2.15	X		2	Ribosome-recycling factor (RRF)	RRF/tRNA synthetase additional domain-like, 145821 [2-184]
Rv2883c	**3NWY**	pyrH	P65929	2.54	X		7	Uridylate kinase (EC 2.7.4.22)	
Rv2911	**2BCF**	dacB; dacB2	Q7D6F2	2.30	X	X	3	D-alanyl-D-alanine carboxypeptidase (EC 3.4.16.4)	
Rv2919c	**3BZQ**	glnB	P64249	1.40			9	Nitrogen regulatory protein P-II	
Rv2925c	**2A11**	rnc	P66666	2.10	X		2	Ribonuclease 3 (EC 3.1.26.3)	
Rv2933	**1PQW**	ppsC	P96202	2.66	X		1	PHENOLPTHIOCEROL SYNTHESIS TYPE-I POLYKETIDE SYNTHASE	
Rv2939	**1Q9J**	papA5	P96208	2.75			1	Phthiocerol/phthiodiolone dimycocerosyl	CoA-dependent acyltransferases, 104590 [1-

								transferase (EC 2.3.1.-)	175], 104591 [181-418]
Rv2941	**3E53**	fadD28	P96290	2.35	X		1	Acyl-CoA synthase (EC 6.2.1.-)	
Rv2945c	**2BYO**	lppX	P65306	2.15	X	X	3	Putative lipoprotein lpp	LolA-like prokaryotic lipoproteins and lipoprotein localization factors, 129497 [20-207]
Rv2965c	**3NBK**	coaD; kdtB	P0A530	1.58	X		3	Phosphopantetheine adenylyltransferase (EC 2.7.7.3)	
Rv2976c	**3A7N**	ung	P67071	1.95	X		2	Uracil-DNA glycosylase (EC 3.2.2.27)	
Rv2981c	**3LWB**	ddl; ddlA	P95114	2.10	X		3	D-alanine--D-alanine ligase (EC 6.3.2.4)	
Rv2986c	**3C4I**	hup; hlp; hupB	P95109	2.04	X		2	DNA-binding protein HU homolog (21 kDa laminin-2-binding protein)	
Rv2987c	**3H5J**	leuD	P65277	1.20	X		7	3-isopropylmalate dehydratase small subunit (EC 4.2.1.33)	
Rv2991	**1RFE**		O53240	2.00	X		10	Conserved hypothetical protein	Split barrel-like, 111796
Rv2992c	**2JA2**	gltX; gltS	P0A636	1.65	X		2	Glutamyl-tRNA synthetase (EC 6.1.1.17)	
Rv2995c	**1W0D**	leuB	P95313	1.65	X		7	3-isopropylmalate dehydrogenase (EC 1.1.1.85)	Isocitrate/Isopropylmalate dehydrogenase-like, 114060
Rv2996c	**1YGY**	serA	P0A544	2.30	X		7	D-3-phosphoglycerate dehydrogenase (EC 1.1.1.95)	Flavodoxin-like, 123151 [3-98], 123151 [283-316]; NAD(P)-binding Rossmann-fold domains, 123150 [99-282]; FwdE/GAPDH domain-like, 123153 [317-451]; Ferredoxin-like, 123152 [452-529]
Rv3014c	**1ZAU**	ligA; lig	P63973	3.15	X		2	DNA ligase (EC 6.5.1.2)	
Rv3020c	**3H6P**	esxS	P64093	1.91	X		3	ESAT-6 LIKE PROTEIN ESXS	
Rv3048c	**1UZR**	nrdF2	Q50549	2.20	X		2	Ribonucleoside-diphosphate reductase subunit beta nrdF2 (EC 1.17.4.1)	Ferritin-like, 108178
Rv3106	**1LQT**	fprA	O05783	1.05	X	X	7	NADPH-ferredoxin reductase (EC 1.18.1.2)	Nucleotide-binding domain, 74199 [2-108], 74199 [325-456]; FAD/NAD(P)-binding domain, 74198 [109-324]
Rv3117	**3AAY**	cysA3; cysA	O05793	1.90	X		7	Putative thiosulfate sulfurtransferase (EC 2.8.1.1)	
Rv3119	**2WP4**	moaE1; moaE	O05795	2.49	X		7	Molybdopterin synthase catalytic subunit (EC 2.-.-.-)	
Rv3132c	**2W3G**	devS; dosS	P95194	1.40	X		9	Redox sensor histidine kinase response regulator (EC 2.7.13.3)	
Rv3133c	**3C57**	devR; dosR	P95193	1.70			9	Transcriptional regulatory protein devR	
Rv3214	**2A6P**	gpm2	Q6MWZ7	2.20	X		7	Possible phosphoglycerate mutase (EC 5.4.2.1)	
Rv3227	**2O0B**	aroA	P22487	1.15	X		7	3-phosphoshikimate 1-carboxyvinyltransferase (EC 2.5.1.19)	
Rv3240c	**1NKT**	secA1	P0A5Y8	2.60	X		3	Protein translocase subunit secA 1 (tbSecA)	P-loop containing nucleoside triphosphate hydrolases, 85832 [-15-225], 85832 [350-396],

									85833 [397-615]; Pre-protein crosslinking domain of SecA, 85830 [226-349]; Helical scaffold and wing domains of SecA, 85831 [616-835]
Rv3246c	**2GWR**	mtrA	C6DXJ2	2.10	X		9	DNA-binding response regulator	
Rv3247c	**1GTV**	tmk	O05891	1.55	X		7	Thymidylate kinase (EC 2.7.4.9)	P-loop containing nucleoside triphosphate hydrolases, 70537
Rv3248c	**3CE6**	ahcY; sahH	P60176	1.60	X		7	Adenosylhomocysteinase (EC 3.3.1.1)	
Rv3250c	**2KN9**	rubB	O05893		X		7	Rubredoxin	
Rv3275c	**3LP6**	purE	P96880	1.70	X		7	N5-carboxyaminoimidazole ribonucleotide mutase (EC 5.4.99.18)	
Rv3279c	**2CGH**	birA	P96884	1.80			7	bifunctional protein: BIOTIN OPERON REPRESSOR + BIOTIN-[ACETYL-COA-CARBOXYLASE] SYNTHETASE (EC 6.3.4.15)	
Rv3280	**2BZR**	accD5; pccB	P96885	2.20			1	propionyl-CoA carboxylase beta chain 5 (PCCase) (EC 6.4.1.3)	
Rv3283	**3HZU**	sseA	P96888	2.10	X		7	thiosulfate sulfurtransferase (EC 2.8.1.1)	
Rv3290c	**2CJG**	lat	P63509	1.95	X		7	Probable L-lysine-epsilon aminotransferase (EC 2.6.1.36)	
Rv3291c	**2W25**	lrp	P96896	2.15			9	Leucine-responsive regulatory protein	
Rv3303c	**1XDI**	lpdA; lpdA-2	O53355	2.81	X		7	NAD(P)H dehydrogenase (EC 1.6.5.2)	FAD/NAD(P)-binding domain, 115160 [2-161], 115160 [276-348]; CO dehydrogenase flavoprotein C-domain-like, 115161 [349-466]
Rv3307	**1G2O**	punA; deoD	P0A538	1.75	X		7	Purine nucleoside phosphorylase (EC 2.4.2.1)	Phosphorylase/hydrolase-like, 60228
Rv3315c	**3IJF**	cdd	O53367	1.99	X		7	Cytidine deaminase (EC 3.5.4.5)	
Rv3356c	**2C2X**	folD	O50385	2.00			7	Bifunctional protein folD [Includes: Methylenetetrahydrofolate dehydrogenase (EC 1.5.1.5); Methenyltetrahydrofolate cyclohydrolase (EC 3.5.4.9)]	
Rv3357	**3D55**		P65067	2.13	X		0	Conserved hypothetical protein; possible antitoxin	
Rv3361c	**2BM5**		O50390	2.00	X		10	Conserved hypothetical proteins	Single-stranded right-handed beta-helix; 128765 [2-182]
Rv3389c	**3KHP**		Q11198	2.30	X		7	MaoC family protein (POSSIBLE DEHYDROGENASE) (EC 1.-.-.-)	
Rv3392c	**1KPG**	cmaA1; cma1	P0C5C2	2.00	X		1	Cyclopropane mycolic acid synthase 1 (EC 2.1.1.79)	S-adenosyl-L-methionine-dependent methyltransferases, 68735
Rv3418c	**1P3H**	cpn10; groES	P09621	2.80	X		0	10 kDa chaperonin (10 kDa antigen)	GroES-like, 87734
Rv3423c	**1XFC**	alr	P0A4X2	1.90	X		7	Alanine racemase (EC 5.1.1.1)	
Rv3462c	**3I4O**	infA	P0A5H5	1.47			2	Translation initiation factor IF-1	

Rv3465	**1UPI**	rmlC; strM	O06330	1.70	X		7	dTDP-4-dehydrorhamnose 3,5-epimerase (EC 5.1.3.13)	Double-stranded beta-helix, 99757
Rv3472	**2CHC**		O06337	1.69			10	Conserved hypothetical protein	Cystatin-like, 146394 [1-167]
Rv3518c	**2XKR**	CYP142	O53563	1.60	X		7	Cholesterol oxidase	
Rv3526	**2ZYL**		P71875	2.30	X		7	POSSIBLE OXIDOREDUCTASE; Rieske [2Fe-2S] domain [PF00355]	
Rv3529c	**2ZQ5**		P71872	2.00			10	Conserved hypothetical protein	
Rv3545c	**3IVY**	cyp125	P63709	1.35	X		7	Putative cytochrome P450 125 (EC 1.14.-.-)	
Rv3568c	**2ZYQ**	bphC	P96850	2.00	X		7	Extradiol ring-cleavage dioxygenase (EC 1.13.11.39)	
Rv3569c	**2WUE**	bphD	P96851	1.80	X		7	2-hydroxy-6-phenylhexa-2,4-dienoic acid hydrolase (EC 3.7.1.-)	
Rv3570c	**3AFF**		P96852	2.00			7	Putative hydroxylase; Acyl-CoA dehydrogenase, C-terminal domain [PF08028]	
Rv3582c	**3OKR**	IspD	3OKR	2.40	X		7	2-C-methyl-D-erythritol 4-phosphate cytidyltransferase	
Rv3588c	**1YM3**	cynT	O53573	1.75	X		7	Carbonic anhydrase (EC 4.2.1.1)	
Rv3592	**3HX9**	TB11.2	O06156	1.75	X		10	Conserved hypothetical protein	
Rv3597c	**2KNG**		P65648				10	Conserved hypothetical protein	
Rv3601c	**2C45**	panD	P65660	2.99			7	Aspartate 1-decarboxylase (EC 4.1.1.11)	
Rv3602c	**3COV**	panC	P0A5R0	1.50	X		7	Pantothenate synthetase (PS) (EC 6.3.2.1)	Adenine nucleotide alpha hydrolase-like, 156877 [3-288]
Rv3607c	**1NBU**	folB	P0A580	1.60	X		7	Probable dihydroneopterin aldolase (EC 4.1.2.25)	T-fold, 91767
Rv3608c	**1EYE**	folP1	P0A578	1.70	X		7	Dihydropteroate synthase 1 (EC 2.5.1.15)	TIM beta/alpha-barrel, 29672
Rv3628	**1SXV**	ppa	P65746	1.30	X		7	Inorganic pyrophosphatase (EC 3.6.1.1)	
Rv3671c	**3K6Y**		O69639	1.30			7	Possible membrane-associated serine protease (EC 3.4.21.-)	
Rv3676	**3D0S**		O69644	2.00	X		9	Transcriptional regulator, Crp/Fnr family	
Rv3710	**3HQ1**	leuA	P96420	1.70	X		7	2-isopropylmalate synthase (EC 2.3.3.13)	
Rv3778c	**3CAI**		P72044	1.80			7	putative aminotransferase (EC 2.6.1.-)	
Rv3803c	**1R88**	mpt51; fbpD	P0A4V6	1.71		X	1	MPT51/MPB51 antigen	alpha/beta-Hydrolases, 97219
Rv3804c	**1SFR**	fbpA; mpt44	P0A4V2	2.70		X	1	Antigen 85-A (EC 2.3.1.-)	alpha/beta-Hydrolases, 105500
Rv3809c	**1V0J**	glf	O06934	2.25	X		3	UDP-galactopyranose mutase (EC 5.4.99.9)	
Rv3846	**1IDS**	sodB; sod; sodA	P17670	2.00	X		0	Superoxide dismutase [Fe] (EC 1.15.1.1)	Long alpha-hairpin, 15722 [2-85]; Fe,Mn superoxide dismutase (SOD), C-terminal domain, 38730 [86-199]
Rv3853	**1NXJ**	rraA; menG	P0A666	1.90	X		2	Regulator of ribonuclease activity A	The "swivelling" beta/beta/alpha domain, 86382

Rv3855	**1T56**	ethR; etaR	P96222	1.70	X		9	HTH-type transcriptional regulator	DNA/RNA-binding 3-helical bundle, 106428 [22-94]; Tetracyclin repressor-like, C-terminal domain, 106429 [95-214]
Rv3874	**1WA8**	esxB; cfp10; lhp	P0A564			X	3	ESAT-6-like protein (10 kDa culture filtrate antigen CFP-10)	Ferritin-like, 120809 [1-99]
Rv3875	**3FAV**	esxA; esaT6	P0A564	2.15	X	X	3	6 kDa early secretory antigenic target (ESAT-6)	
Rv3913	**2A87**	trxB	P52214	3.00	X		7	Thioredoxin reductase (EC 1.8.1.9)	
Rv3914	**2I1U**	trxA; trx; trxC	P0A616	1.30			7	Thioredoxin	

a) According to Wilmanns and Kaufmann (11).

b) Classification according to Camus *et al*. (13).

c) Number following fold name is the SCOP accession number (http://scop.mrc-lmb.cam.ac.uk/scop/). Number in brackets is the amino acid numbering of the domains possessing that fold. Entries without a given fold have not been annotated in the SCOP database.

If more than one structure for a ORF is available, that with the highest resolution is given in the Table. Other structures can be found in the PDB using similarity search tools (http://www.rcsb.org/pdb/home/home.do). Those structures that contain ligands, either natural cofactors, substrates, bound metal ions, or substrate analogs are indicated. Entries which are not annotated as containing ligands may have a related structure in the PDB that does have bound ligands. Numbers in square brackets under the heading *Functional annotation* are PFam accession numbers and might provide some insight into the possible function of uncharacterized proteins. Full references for each entry can be found in the PDB. Lig., Ligands; ORF, open reading frame; PDB, Protein Data Bank; Res., Resolution; Sec., Secreted.
